# Exercise before Breakfast

**Published:** 1871-02

**Authors:** 


Exercise before Breakfast.—At least an hour should elapse after rising before breakfast. It is better to take some light exercise. The appetite will be better, and the food will digest easier and be more easily assimilated.—*Herald of Health.*

